# Association between mortality and serum uric acid levels in non-diabetes-related chronic kidney disease: An analysis of the National Health and Nutrition Examination Survey, USA, 1999–2010

**DOI:** 10.1038/s41598-020-74747-w

**Published:** 2020-10-16

**Authors:** Chia-Lin Lee, Shang-Feng Tsai

**Affiliations:** 1 Division of Endocrinology and Metabolism, Department of Internal Medicine, Taichung Veterans General Hospital, Taichung, Taiwan; 2 Department of Medical Research, Taichung Veterans General Hospital, Taichung, Taiwan; 3 Department of Public Health, College of Public Health, China Medical University, Taichung, Taiwan; 4 School of Medicine, National Yang-Ming University, Taipei, Taiwan; 5 Division of Nephrology, Department of Internal Medicine, Taichung Veterans General Hospital, 160, Sec. 3, Taiwan Boulevard, Taichung, 407 Taiwan; 6 Department of Life Science, Tunghai University, Taichung, Taiwan

**Keywords:** Biomarkers, Nephrology, Risk factors

## Abstract

The relationship between serum uric acid (SUA) and cardiovascular (CV) mortality in patients with chronic kidney disease (CKD) has been described as either a J- or U-shaped function. However, its effect in non-diabetic CKD (and varying severities of CKD) remains unclear. We analyzed the database of the National Health and Nutrition Examination Survey, USA, from the years 1999 to 2010. We then grouped the subjects into 4 categories according to their SUA levels: (a) < 5 mg/dl, (b) 5–7 mg/dl, (c) 7–9 mg/dl and (d) ≥ 9 mg/dl. For mortality comparison purposes (CV related, cancer related and all-cause mortality), we set the SUA group of 5–7 mg/dl as the reference. We also separated this population into moderate (stage 3) and severe (stages 4 and 5) CKD. A total of 1860 participants were included in this study. Results showed that the group with the lowest SUA levels (< 5 mg/dl), were the least male gender (19.25%), had the lowest body mass index (26.41(95% CI = 25.66–27.16) kg/m^2^), highest systolic blood pressure (139.02 (95% CI 135.72–142.32) mmHg), highest high-density cholesterol (59.55 (95% CI 57.37–61.74) mg/dl), lowest blood glucose (95.46 (95% CI 93.16–97.76) mg/dl), highest total cholesterol (210.31 (95% CI 203.36–217.25) mg/dl), lowest serum albumin (4.09 (95% CI 4.04–4.14) g/dl), highest estimated glomerular filtration rate (eGFR) (47.91 (95% CI 45.45–50.49) ml/min/1.732m^2^), least history of hypertension (54.4%), and least total energy intake (1643.7 (95% CI 1536.13–1751.27) kcal/day). In the group with SUA ≥ 9 mg/dl, patients had higher all-cause mortality (HR = 2.15) whatever their baseline CVD status. In non-DM CKD patients with a CVD history, the group with SUA ≥ 9 mg/dl had the highest all-cause mortality (HR = 5.39), CVD mortality (HR = 8.18) and CVD or cancer (HR = 8.25) related mortality. In non-DM patients with severe CKD (eGFR < 30 ml/min/1.732m^2^), the group with SUA < 5 had a significantly increased all-cause mortality. On the contrary, in non-DM patients with moderate CKD (eGFR = 30–60 ml/min/1.832m^2^), the group with SUA ≥ 9 had a significantly increased all-cause mortality. In moderate non-DM CKD, SUA ≥ 9 mg/dl is associated with higher all-cause mortality. However, once progressing to severe non-DM CKD, SUA < 5 mg/dl is associated with higher all-cause mortality (even though it has the least risk factors for metabolic syndrome).

## Introduction

High serum uric acid (SUA) levels predict myocardial infarction, a finding that was first reported in 1951^1^. Since then, epidemiological studies have further shown positive associations between high SUA levels and cardiovascular disease (CVD) related mortality^2–5^ with a possible causal effect. However, other studies have reported contradicting evidence on such causal association^6–8^. According to Band et al.^6^, their study reports that SUA does not predict CVD by using multivariate analysis (included age, systolic blood pressure (SBP), body weight, cigarette smoking, and serum cholesterol). There is still no consensus regarding this causal association between SUA and CVD related mortality. Until now, only those guidelines followed in Japan recommend that treatment of asymptomatic hyperuricemia be used in order to obtain cardiovascular and renal benefits^9^. Such conflicting results are in part due to differences in baseline risk factors surrounding atherosclerosis (such as pre-existing CVD, diuretic treatment^7^, obesity^10^, hypertension^11^ and diabetes mellitus (DM)^12^).

Notably, a positive correlation is found between creatinine clearance and 24-h UA excretion (r = 0.61), as well as similarly between creatinine clearance and filtered UA load (r = 0.82)^12^. Reasonably, the adjustment for baseline renal functions in those studies was needed for their outcome analyses. No reported study has been conducted to differentiate the association between SUA and mortality according to different severities of CKD. In addition, SUA may be a risk factor for CVD through mechanisms such as atherosclerosis and insulin resistance. Therefore, the baseline confounding factors for atherosclerosis should also be adjusted, particularly when concerning the condition of DM. To the best of our knowledge, most studies have focused on the association between mortality and SUA in patients with DM (without or without chronic kidney disease (CKD)). Limited studies are available regarding this outcome in patients without DM. Also, causes of high mortality may be different between those with hyperuricemia and hypouricemia. At last, DM is the leading cause of end-stage renal disease (ESRD), however in more than half of the cases, the consequence are not related to DM. The aim of the present study was to investigate the predictive role of different levels of SUA on mortality (including CVD and those cancer related) in patients with various different severities of non-diabetic CKD.

## Methods

### Study population and data collection

#### National health and nutrition examination survey (NHANES)

The National Health and Nutrition Examination Survey (NHANES) is one of a series of health-related programs in the USA conducted periodically by the Centers for Disease Control (CDC) and Prevention’s National Center for Health Statistics (NCHS), which release their data to the public. The Research Ethics Review Board at the NCHS approved our survey protocol, and all participants or proxies provided written informed consent. This large ongoing dietary survey was conducted in order to assess the health and nutritional status of community-dwelling individuals in the USA cross-sectionally. The examinations included anthropometric measurements, questionnaires on health and nutrition, along with laboratory testing. All participants completed in-home interviews. We analyzed participants in the NHANES from the years 1999 to 2010. Participants were excluded from analyses if they were < 18 years of age, had no data on their estimated glomerular filtration rate (eGFR), had incomplete data with respect to anthropometric measurements, questionnaires, or laboratory tests.

For histories of DM, CHD (coronary heart disease) and stroke, questionnaires were completed in order to collect data: MCQ160C for CHD, MCQ220 for cancer or malignancy, MCQ160F for stroke and DIQ010 for NCHS and NHAES^13^. These surveys were created to record any match between NHANES and National Death Index (NDI) death certificate records, which is an NCHS centralized database of all deaths in the USA from 1979 onward. DM was defined as being self-reported-after a physician’s diagnosis of DM or the self-reported mention of taking insulin or anti-diabetic pills. The participants with CHD or who had a stroke were defined by their answer to the question: "Have you ever been told you had CHD or a stroke?”. We defined cardiovascular disease (CVD) as either CHD or stroke.

### Mortality outcomes

Mortality outcomes of interest include all-cause mortality, CVD death, and cancer death. The causes of death were based upon the ICD-10 (10th revision of the International Statistical Classification of Diseases and Related Health Problems) codes defined in the NHANES Public-use Linked Mortality Files^14^, which contain mortality follow-up data on NHANES participants linked via National Death Index (NDI) death certificate records (NCHS 2011 Linked Mortality Files Matching Methodology. National Center for Health Statistics, Office of Analysis and Epidemiology; Hyattsville, MD, USA: 2013^15^). The underlying causes of death were coded using the UCOD_LEADING variable and classified as either all-cause deaths or deaths due to CVD (UCOD_LEADING values 001 Diseases of the heart or 005 Cerebrovascular diseases), as well as cancer mortality (UCOD_LEADING value 002 Malignant neoplasms). These specific codes were as follows: codes of C00–C97 were categorized as death from malignant neoplasms (cancer death), while the codes of I00–I09, I11, I13, I20–I51, and I60–I69 were categorized as cardiovascular death. We followed the participants through December 31 2011.

### Other data definitions

Baseline variables included the following variables: age, gender, body mass index (BMI), estimated glomerular filtration rate (eGFR)(ml/min/1.732m^2^)^16^, systolic blood pressure (SBP) (mmHg), and diastolic blood pressure (DBP), total cholesterol (TC) (mg/dl), high-density lipoprotein (HDL) (mg/dl), serum creatinine (SCr) (mg/dl) and fasting plasma glucose (mg/dl). Hypertension was defined as being self-reported after a physician’s diagnosis of hypertension or being self-reported due to taking anti-hypertensive pills. SCr measurements were recalibrated to the standard SCr measurements obtained at the Cleveland Clinic Research Laboratory (Cleveland, Ohio)^17^. We calculated the estimated glomerular filtration rate (eGFR) by using the Chronic Kidney Disease Epidemiology Collaboration (CKD-EPI) equation (ml/min/1.732m^2^)^16^. The definition of CKD was based upon the international guideline group Kidney Disease Improving Global Outcomes (KDIGO) in 2004^18–20^. We only used eGFR < 60 ml/min/1.732m^2^ for the definition of CKD. Different severities of CKD included moderate CKD at 30–60 ml/min/1.732m^2^ of eGFR, and severe CKD at < 30 ml/min/1.732m^2^ of eGFR. Our study was approved by the Human Research Review Committee of Taichung Veterans General Hospital (CE18312A). Regarding ethics approval and consent to participate: this study was approved by the Ethics Committee of Taichung Veterans General Hospital, IRB number:CE18312A. Additionally, all studies were performed in accordance with the relevant guidelines and regulations.

### Statistical analyses

Hyperuricemia was labeled as SUA levels exceeding 7 mg/dl. The treatment target for SUA in patients with gout was to control at such levels < 6 mg/dl, and if coexisting with renal UA stones or tophi, < 5 mg/dl^21^. Therefore, we divided the subjects into 4 groups according to their SUA levels : (a) < 5 mg/dl, (b) 5–7 mg/dl, (c) 7–9 mg/dl and (d) ≥ 9 mg/dl. For mortality comparison purposes, we set the group with SUA levels of 5–7 mg/dl as the reference, which is in line with the available literature^22–26^.

Analysis of Variance (ANOVA) and *Chi*-square tests were used to examine the differences in baseline demographics and characteristics across different levels of SUA. Continuous data were presented as mean ± 95% CI (confidence interval), unless stated otherwise. Due to the distribution of creatinine and eGFR did not follow normal distribution, geometric means are reported. We utilized Cox proportional hazards regression models to compare the hazard ratios (HRs) and 95% CI (confidence interval) for all-cause, CV, and cancer mortality across different SUA levels. Naturally, history of established CVD is a known risk factor regarding CVD mortality^27^. Therefore, subgroup analysis was also separately performed in participants who were both with and without a history of CVD. We did the restricted cubic spline as sensitivity test. First, we checked the curvature (non-linear relationship) between continuous SUA level and different outcomes. Five knots (5, 27.5, 50, 72.5 and 95 percentiles of exposure) were used for restricted cubic spline modeling. SUA of 7 mg/dl was set as reference point. If p value for non-linear relationship less than 0.05, we will plot the restricted cubic spline. Otherwise, if we cannot select any spline variables with outcomes, we will test the linear relationship between SUA and outcomes.

Moreover, because approximately 70% of SUA is excreted from the kidneys, hyperuricemia was mostly noticed as renal function deterioration^28^. We further performed the separate analysis through different eGFR categories (30–60 ml/min/1.732m^2^ of eGFR and < 30 ml/min/1.732m^2^ of eGFR). In addition, age, gender, race/ethnicity, BMI, HDL-C, hypertension, smoking status, and hemoglobin A1C (HbA1C) were adjusted in all survival analyses.

The study design of NHANES is a complex survey design. All analyses need to be adequately weighted in order to properly represent the US population. We calculated the weighted data according to analytic guidelines [National Health and Nutrition Examination Survey: Analytic Guidelines, 2011–2014 and 2015–2016 (accessed on 29 October 2019)^29^. All analyses were performed using the Statistical Analysis System survey procedures (SAS version 9.4, 2013, Cary, NC, USA). All p-values < 0.05 were considered statistically significant.

### Ethics approval and consent to participate

This study was approved by the Ethics Committee of Taichung Veterans General Hospital, IRB number:CE18312A. All methods were performed in accordance with the relevant guidelines and regulations.

### Consent for publication

All authors all agree on publication in this journal.

## Results

**Figure 1:**
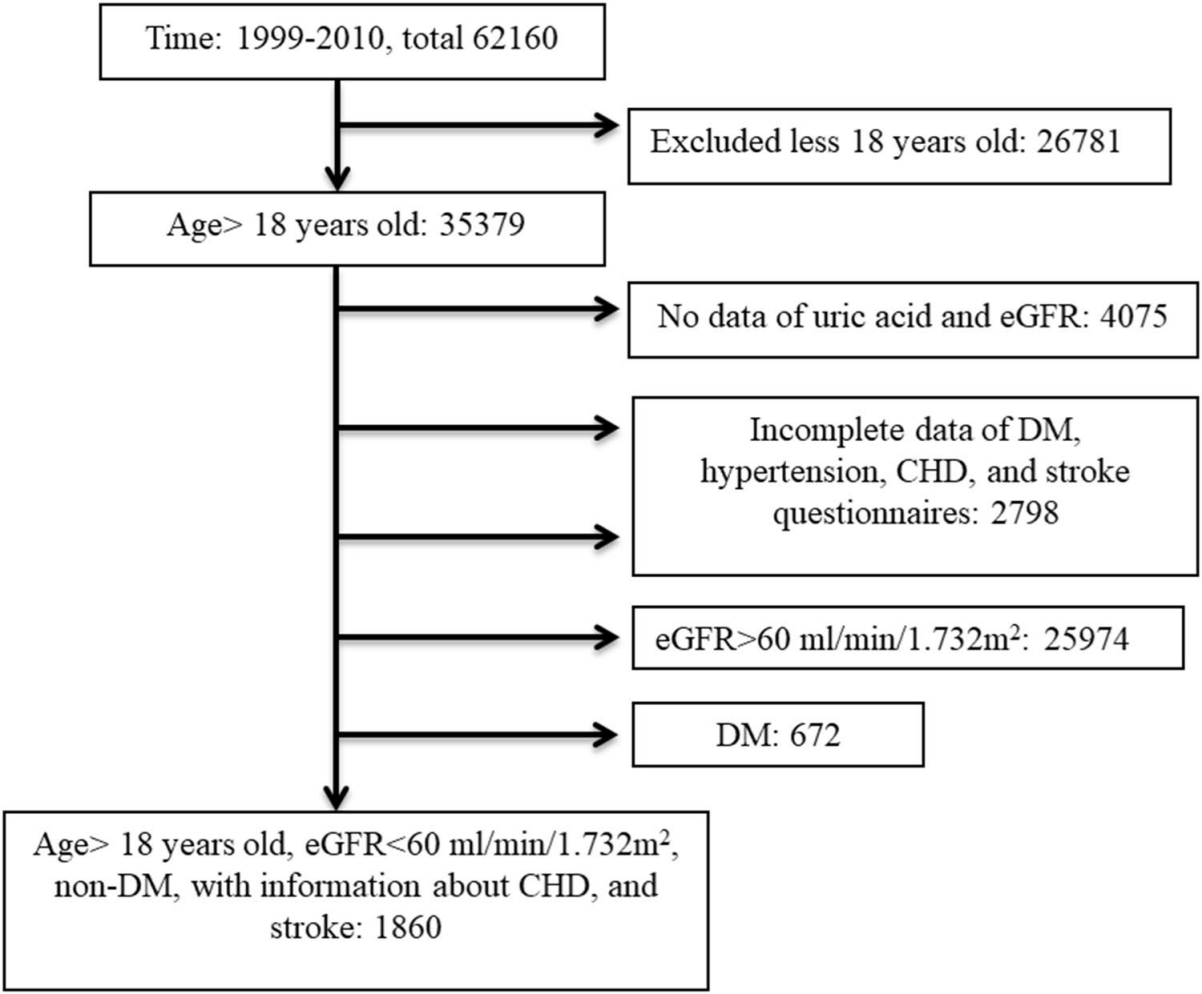
Flow diagram for selection of participants.

We analyzed participants in the NHANES from the years 1999 to 2010. Initially, a total 62,160 people were screened. We first excluded those of a younger age (< 18 years old), those missing data regarding UA, non-CKD patients (> 60 ml/min/1.732m^2^ of eGFR), those with no information of CHD or stroke, and no information of DM. Finally, we included 1860 participants (those with non-DM related CKD, with data regarding SUA and a history or not a history of CVD) for this study (Fig. 1). Their baseline conditions are shown in Table 1. Most patients (47.0%) had hyperuricemia (5–7 mg/dl of SUA). Only 278 patients (14.9%) had the lowest SUA level (< 5 mg/dl), while 133 patients (7.2%) had the highest SUA level (≥ 9 mg/dl). In the group with the lowest SUA levels, most of them had the lowest BMI (26.41 (95% CI 25.66–27.16) kg/m^2^), highest SBP (139.02 (95% CI 135.72–142.32) mmHg), highest HDL (59.55 (95% CI 57.37–61.74) mg/dl), lowest blood glucose (95.46 (95% CI 93.16–97.76) mg/dl), highest total cholesterol (210.31 (95% CI 203.36–217.25) mg/dl), lowest serum albumin (4.09 (95% CI 4.04–4.14) g/dl), highest eGFR (47.91 (95% CI 45.45–50.49) ml/min/1.732m^2^), least history of hypertension (54.4%), and least total energy intake (1643.7(95% CI 1536.13–1751.27) kcal/day). With increasing SUA, patients were more male gender (p < 0.001), had a higher BMI (p < 0.001), lower HDL (p < 0.001), higher blood glucose (p < 0.001), lower eGFR (p < 0.001) and more history of hypertension (p < 0.001). In summary, patients in the group with UA < 5 mg/dl had the least risk factors for CVD according to the Framingham Heart Study^30^: mostly female gender, had the lowest BMI, highest HDL, lowest blood glucose, and least history of hypertension. Additionally, they had best renal function in all of the four groups.

**Table 1:** Baseline characteristics of non-DM related CKD.

Uric acid (mg/dl)	All	< 5	5–7	7–9	> 9	P value
N (%)	1860	278 (14.9)	874 (47.0)	575 (30.9)	133 (7.2)	
Age, years	72.04 (71.17–72.91)	72.23 (70.62–73.85)	72.64 (71.42–73.85)	71.19 (69.76–72.62)	70.85 (67.57–74.13)	< 0.001
Male (%)	876 (40)	70 (19.25)	385 (36.89)	326 (49.97)	95 (64.66)	< .0001
Non-Hispanic black (%)	264 (7.6)	29 (6.1)	103 (6.06)	101 (9.63)	31 (13.85)	0.0004
Current smoke (%)	183 (21)	18 (17.51)	95 (24.09)	55 (18.96)	15 (16.2)	0.3541
Cancer (%)	431 (23)	64 (23.2)	204 (23.69)	138 (23.29)	25 (20.57)	0.9383
BMI, kg/m^2^	28.04 (27.79–28.29)	26.41 (25.66–27.16)	27.66 (27.24–28.09)	29.16 (28.64–29.69)	29.96 (28.64–31.29)	< 0.001
SBP	137.25 (138.89-138.62)	139.02 (135.72-142.32)	137.38 (135.54-139.21)	137.35 (134.75-139.95)	131.06 (125.5-136.62)	< 0.001
HDL-C, mg/dl	53.84 (52.91-54.77)	59.55 (57.37-61.74)	54.97 (53.57-56.38)	50.24 (48.71-51.77)	46.99 (43.87-50.11)	< 0.001
Glucose Serum, mg/dl	98.82 (97.69-99.95)	95.45 (93.16-97.76)	98.34 (96.54-100.16)	100.49 (98.62-102.37)	103.43 (98.4-108.46)	< 0.001
Total cholesterol, mg/dl	202.33 (199.69-207.97)	210.31 (203.36-217.25)	201.57 (198.21-204.94)	199.68 (195.42-203.93)	200.46 (190.5-210.42)	< 0.001
Serum creatinine (mg/dL)	1.31 (1.29-1.34)	1.20 (1.15-1.26)	1.29 (1.25-1.33)	1.36 (1.33-1.40)	1.63 (1.26-1.72)	< 0.001
Albumin (g/dL)	4.14 (4.12-4.16)	4.09 (4.04-4.14)	4.14 (4.11-4.18)	4.18 (4.14-4.21)	4.13 (4.03-4.22)	< 0.001
eGFR	45.8 (44.87-46.76)	47.9 (45.45-50.49)	46.3 (44.88-47.72)	45.6 (44.39-46.80)	38.5 (35.93-41.18)	< 0.001
History of hypertension (%)	1242 (65)	159 (54.44)	549 (61.71)	430 (72.6)	104 (77.21)	< 0.0001
Total energy (kcal/day)	1716.78 (1670.46–1763.11)	1643.7 (1536.13–1751.27)	1715.78 (1651.67–1779.9)	1755.68 (1678.76–1832.61)	1726.46 (1561.86–1891.05)	< 0.001

Adjust for BMI, gender, age (month), race, HDL-Cholesterol (mg/dL), current smoke, SBP, history of hypertension, HbA1C.

Detail information of mortality according to four levels of SUA was shown as case numbers and percentage per 1000 person-year in supplementary Table 1. The results of mortality analysis (all-cause, CVD and cancer related mortality) are shown in Table 2 according to baseline CVD or not. In the group with SUA ≥ 9 mg/dl (when compared with the reference group, i.e., SUA of 5–7 mg/dl), patients had higher all-cause mortality (HR = 2.15) whatever their baseline CVD status. In non-DM CKD patients without a CVD history, there was no statistical increase or decrease in mortality. However, in non-DM CKD patients with a CVD history, the group with SUA ≥ 9 mg/dl had the highest all-cause mortality (HR = 5.39), CVD mortality (HR = 8.18) and CVD or cancer (HR = 8.25) related mortality. Curves (all-cause, CVD and cancer related) of mortality according to different levels of SUA were plotted in Supplementary Figs. 1A (with/without baseline CVD), 1B (without baseline CVD) and 1C (with baseline CVD). All detailed 12 curves (non-linear relationship) between continuous SUA and different outcomes were also plotted in Supplementary Fig. 2.

**Table 2:** Relative HRs (95% CI) of mortality in non-DM related CKD (with or without CVD).

Uric acid (mg/dl)	< 5	5–7	7–9	> 9
**With or without CVD history**				
All-cause mortality	1.066 (0.582–1.951)	1.0 (reference)	1.238 (0.839–1.826)	**2.149 (1.128–4.095)**
CVD mortality	1.475 (0.538–4.043)	1.0 (reference)	1.199 (0.618–2.326)	2.308 (0.856–6.228)
Cancer mortality	0.857 (0.189–3.879)	1.0 (reference)	1.234 (0.509–2.99)	1.877 (0.532–6.625)
CVD or cancer death	1.21 (0.51–2.871)	1.0 (reference)	1.21 (0.709–2.067)	2.111 (0.983–4.533)
**Without CVD history**				
All-cause mortality	0.939 (0.501–1.758)	1.0 (reference)	1.346 (0.796–2.278)	1.615 (0.703–3.708)
CVD mortality	1.355 (0.352–5.207)	1.0 (reference)	1.27 (0.46–3.505)	0.841 (0.134–5.3)
Cancer mortality	0.406 (0.05–3.318)	1.0 (reference)	1.279 (0.465–3.521)	1.474 (0.392–5.539)
CVD or cancer death	0.847 (0.285–2.515)	1.0 (reference)	1.305 (0.643–2.648)	1.093 (0.357–3.343)
**With CVD history**				
All-cause mortality	1.43 (0.495–4.134)	1.0 (reference)	1.03 (0.596–1.781)	**5.387 (2.512–11.549)**
CVD mortality	2.197 (0.419–11.513)	1.0 (reference)	1.036 (0.445–2.414)	**8.183 (2.212–30.27)**
Cancer mortality	4.266 (0.421–43.224)	1.0 (reference)	1.163 (0.196–6.9)	9.176 (0.98–85.903)
CVD or cancer death	2.735 (0.74–10.105)	1.0 (reference)	1.086 (0.55–2.143)	**8.248 (2.93–23.224)**

Adjust for BMI, gender, age (month), race, HDL-Cholesterol (mg/dL), current smoke, SBP, history of hypertension, HbA1C.

**Figure 2:**
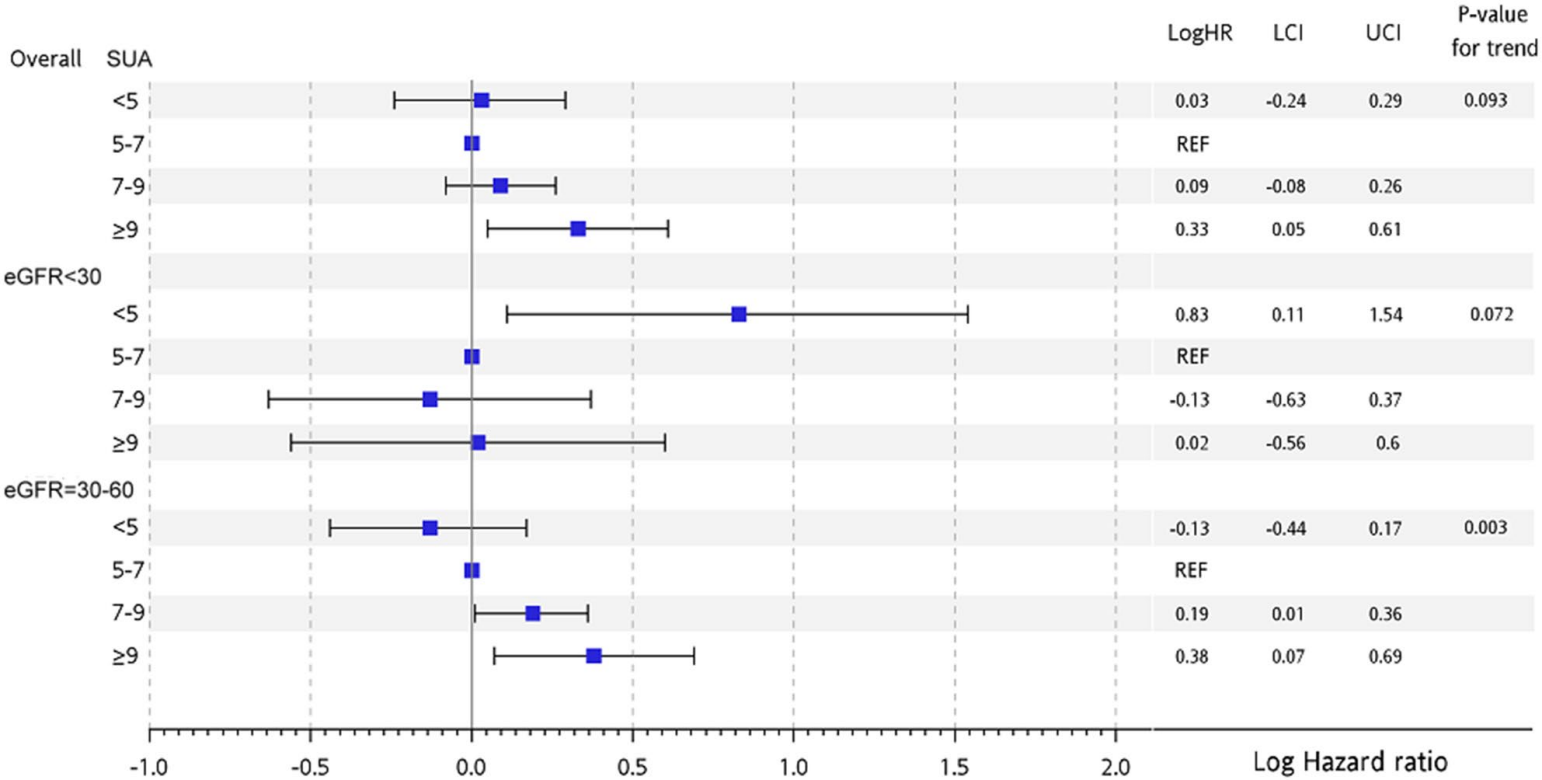
Hazard ratio of all-cause mortality according to different levels of eGFR.

The all-cause mortality was significantly increased with baseline CVD. Further analysis according to varying severities of CKD is shown in Fig. 2. In non-DM patients with severe CKD (eGFR < 30 ml/min/1.732m^2^), the group of SUA < 5 experienced a significantly increased all-cause mortality. However, in non-DM patients with moderate CKD (eGFR = 30–60 ml/min/1.832m^2^), the group of SA ≥ 9 showed a significantly increased all-cause mortality.

## Discussion

DM is the leading cause of atherosclerotic cardiovascular disease (ASCVD), heart failure (HF), CKD and ESRD. However, at least half of the causes of CKD are non-DM related. With the progression of renal functions, CV outcomes (including CVD mortality, re-infarction, CHF, stroke, resuscitation and composite end-point) all get worse. Both CVD and all-cause mortality are high in CKD cases regardless of whether its condition is DM related or not^31^. The increase in CVD or all-cause mortality can be seen in both DM and non-DM related CKD^31^. In 1951, high SUA levels were reported as a means to predict myocardial infarction^1^. More epidemiological evidence has since been made available relating high SUA and CHD related mortality^2–5^ and the relationship may even be causal. Recently, high SUA was found to be an independent risk factor for CVD mortality^32^. However, other studies have found contradicting results^6–8,33,34^. According to the most recent meta-analysis for CKD patients published in 2019^3^, patients in the group with the highest SUA levels are associated with an increased risk of CVD mortality when compared with patients experiencing the lowest levels (HR = 1.47, 95% CI 1.11–1.96). Noticeably, there is some degree of heterogeneity in that meta-analysis. Possible explanations for the conflicting results are the differences in baseline risk factors for atherosclerosis, including pre-existing CHD, diuretic treatment^7^, obesity^10^, hypertension^11^ and DM^12^, since SUA is also a risk factor for atherosclerosis. None of the preceding studies displayed good control over the confounding factors for metabolic syndromes, particularly whether DM was involved or not. The incidence of DM ranges from 5 to 59% in CKD patients according a meta-analysis^3^. Our study is the first to evaluate the association between SUA and mortality in non-DM related CKD.

Verdecchia et al., reported that SUA levels < 4.5 mg/dl in men or < 3.2 mg/dl in women are associated with higher risks of CVD^35^. Mazza et al., reported that elderly subjects with SUA levels < 4.9 mg/dl have a higher CVD mortality in those diagnosed with type 2 DM^36^. SUA was an independent predictor for the risk of CHD mortality in a J-shaped manner^36^. In another prospective cohort study^25^, levels of SUA ≥ 8 or < 4 mg/dL predicted all-cause and CVD-related mortalities independently in the elderly, with the relationship plotted in the form of a U-shaped function, and being particularly clear in those subjects who were undernourished. The relationship between SUA levels and CV mortality is often viewed as not being an issue of “the lower the better”^22–25,37^. However, none of the above studies focus on pure non-DM related CKD, nor do they differentiate the association between mortality and SUA in varying severities of CKD. To best of our knowledge, our study is the first to research the association between mortality and different levels of SUA regarding varying severities of non-DM related CKD. In moderate non-DM CKD, a level of SUA ≥ 9 mg/dl is associated with higher all-cause mortality. However, once progressing to severe non-DM CKD, a level of SUA < 5 mg/dl is associated with higher all-cause mortality.

In severe non-DM CKD, a level of SUA < 5 mg/dl is associated with higher all-cause mortality. Since most SUA is excreted by the kidneys^12^, the SUA should be higher with the deterioration of renal function. According to Tae el al, most SUA levels are values of quartile 3 and 4^38^. Thus, in patients with severe CKD but with levels of SUA < 5 mg/dl, their SUA was reduced the most when compared to other groups. Many reasons can explain the higher all-cause mortality in patients with non-DM CKD and SUA < 5 mg/dl. There are many physiological functions surrounding SUA^39^. First, more than 50% of the antioxidant capacity of plasma comes from SUA^40,41^. Particularly in vascular endothelial cells and human nasal secretions, SUA was responsible for an antioxidant^42,43^. Second, SUA is vital for tissue healing through an inflammatory process, while also mobilizing progenitor endothelial cells^44^. Another study has shown that extremely low SUA (loss-of-function mutations of SLC22A12 encoding blood vessels) would cause endothelial dysfunction in vivo^45^. Third, SUA is also associated with being a mediator of type 2 immune responses^46^, while also providing defense against neurological and autoimmune diseases^47^. The extremely reduced SUA in patients with severe CKD may affect many physiological function of SUA, particularly the endothelial function. That is the reason patients in the group with SUA < 5 mg/dl experienced the fewest risk factors of CVD according to the Framingham Heart Study (least umber of male gender, lowest BMI, highest HDL, lowest blood glucose, and least history of hypertension), still had significantly higher all-cause mortality and CVD related mortality. In addition to the compromised physiological function of SUA in endothelium, there still remain novel or uremic related risk factors causing CVD with medial calcification (arteriosclerosis)^48^. Both CKD related risk factors and traditional risk factors (for metabolic syndrome) can lead to vascular stenosis, media and intima, respectively^48^. Even with having the least amount of risk for metabolic syndrome in this group (SUA < 5 mg/dl), patients still experienced poor renal function, which caused severe medial calcification (arteriosclerosis) related CVD and all-cause mortality. Finally, the lowest SUA may be due to malnutrition. Patients in the group of SUA < 5 mg/dl had the lowest total energy intakes (1643.7 (95% CI 1536.13–1751.27) kcal (p < 0.001)), and lowest serum albumin (4.09 (95% CI 4.04–4.14) g/dl, p < 0.001), therefore having the lowest BMI across the various groups. Extremely reduced SUA in patients with severe CKD suggests malnutrition, which is reported to be associated with higher mortality^49^. Malnutrition-inflammation-atherosclerosis syndrome (MIA) is known as being a notorious complex condition in CKD patients^50^.

This study has some limitations. First, we received no information regarding medication (particularly SUA-lowering agents). Second, we have no other markers regarding the nutritional status (serum albumin or subjective global assessment) of the patients. Third, detailed causes of death were not available. Fourth, we did not have data surrounding physical activity and metabolic equivalent of task as physical activity may be associated with mortality. Finally, the definition of CKD was only eGFR-based in this study, and albuminuria was not included. However, our study has the merit of being the first study of its kind for non-DM related CKD patients when exploring the association between SUA and mortality.

## Conclusion

In this study, the association between SUA and mortality for varying severities of non-DM related CKD was analyzed for the first time. In moderate non-DM CKD, a level of SUA ≥ 9 mg/dl is associated with higher all-cause mortality. However, once progressing to severe non-DM CKD, a level of SUA < 5 mg/dl is associated with higher all-cause mortality.

## Supplementary information

Supplementary file1

## Abbreviations

BMI: Body mass index
CDC: Centers for disease control
CKD: Chronic kidney disease
CKD-EPI: Chronic kidney disease epidemiology collaboration
CHD: Coronary heart disease
CV: Cardiovascular
DM: Diabetes mellitus
DBP: Diastolic blood pressure
ESRD: End-stage renal disease
HDL: High-density lipoprotein
NCHS: National Center for Health Statistics
NDI: National death index
NHANES: National health and nutrition examination survey
SCr: Serum creatinine
SBP: Systolic blood pressure
SUA: Serum uric acid
TC: Total cholesterol
