# Author Correction: Association between mortality and serum uric acid levels in non-diabetes-related chronic kidney disease: An analysis of the National Health and Nutrition Examination Survey, USA, 1999–2010

**DOI:** 10.1038/s41598-021-97179-6

**Published:** 2021-09-01

**Authors:** Chia-Lin Lee, Shang-Feng Tsai

**Affiliations:** 1 Division of Endocrinology and Metabolism, Department of Internal Medicine, Taichung Veterans General Hospital, Taichung, Taiwan; 2 Department of Medical Research, Taichung Veterans General Hospital, Taichung, Taiwan; 3 Department of Public Health, College of Public Health, China Medical University, Taichung, Taiwan; 4 School of Medicine, National Yang-Ming University, Taipei, Taiwan; 5 Division of Nephrology, Department of Internal Medicine, Taichung Veterans General Hospital, 160, Sec. 3, Taiwan Boulevard, Taichung, 407 Taiwan; 6 Department of Life Science, Tunghai University, Taichung, Taiwan

Correction to: *Scientific Reports* 10.1038/s41598-020-74747-w, published online 16 October 2020

The original version of this Article contained errors in Table 1, where the data values were incorrectly given for “SBP”, “HDL-C, mg/dl”, “Glucose Serum, mg/dl”, “Total cholesterol, mg/dl”, “Serum creatinine (mg/dL)”, “Albumin (g/dL)”, and “eGFR”. The correct and incorrect values appear below.

Incorrect:

**Table:** 

Uric acid (mg/dl)	All	< 5	5-7	7-9	≥9	P value
SBP	28.04 (27.79–28.29)	26.41 (25.66–27.16)	27.66 (27.24–28.09)	29.16 (28.64–29.69)	29.96 (28.64–31.29)	< 0.001
HDL-C, mg/dl	28.04 (27.79–28.29)	26.41 (25.66–27.16)	27.66 (27.24–28.09)	29.16 (28.64–29.69)	29.96 (28.64–31.29)	< 0.001
Glucose Serum, mg/dl	28.04 (27.79–28.29)	26.41 (25.66–27.16)	27.66 (27.24–28.09)	29.16 (28.64–29.69)	29.96 (28.64–31.29)	< 0.001
Total cholesterol, mg/dl	28.04 (27.79–28.29)	26.41 (25.66–27.16)	27.66 (27.24–28.09)	29.16 (28.64–29.69)	29.96 (28.64–31.29)	< 0.001
Serum creatinine (mg/dL)	28.04 (27.79–28.29)	26.41 (25.66–27.16)	27.66 (27.24–28.09)	29.16 (28.64–29.69)	29.96 (28.64–31.29)	< 0.001
Albumin (g/dL)	28.04 (27.79–28.29)	26.41 (25.66–27.16)	27.66 (27.24–28.09)	29.16 (28.64–29.69)	29.96 (28.64–31.29)	< 0.001
eGFR	28.04 (27.79–28.29)	26.41 (25.66–27.16)	27.66 (27.24–28.09)	29.16 (28.64–29.69)	29.96 (28.64–31.29)	< 0.001

Correct:

**Table:** 

Uric acid (mg/dl)	All	< 5	5-7	7-9	≥ 9	P ﻿valu﻿e
SBP	137.25 (138.89–138.62)	139.02 (135.72–142.32)	137.38 (135.54–139.21)	137.35 (134.75–139.95)	131.06 (125.5–136.62)	< 0.001
HDL-C, mg/dl	53.84 (52.91–54.77)	59.55 (57.37–61.74)	54.97 (53.57–56.38)	50.24 (48.71–51.77)	46.99 (43.87–50.11)	< 0.001
Glucose Serum, mg/dl	98.82 (97.69–99.95)	95.45 (93.16–97.76)	98.34 (96.54–100.16)	100.49 (98.62–102.37)	103.43 (98.4–108.46)	< 0.001
Total cholesterol, mg/dl	202.33 (199.69–207.97)	210.31 (203.36–217.25)	201.57 (198.21–204.94)	199.68 (195.42–203.93)	200.46 (190.5–210.42)	< 0.001
Serum creatinine (mg/dL)	1.31 (1.29–1.34)	1.20 (1.15–1.26)	1.29 (1.25–1.33)	1.36 (1.33–1.40)	1.63 (1.26–1.72)	< 0.001
Albumin (g/dL)	4.14 (4.12–4.16)	4.09 (4.04–4.14)	4.14 (4.11–4.18)	4.18 (4.14–4.21)	4.13 (4.03–4.22)	< 0.001
eGFR	45.8 (44.87–46.76)	47.9 (45.45–50.49)	46.3 (44.88–47.72)	45.6 (44.39–46.80)	38.5 (35.93–41.18)	< 0.001

The original Article has been corrected.

