# Supplementary material for: Association between mortality and serum uric acid levels in non-diabetes-related chronic kidney disease: An analysis of the National Health and Nutrition Examination Survey, USA, 1999–2010
Source: Sci Rep. 2020 Oct 16;10:17585. doi: 10.1038/s41598-020-74747-w (PMC7568534; doi:10.1038/s41598-020-74747-w)

Association between mortality and serum uric acid levels in non-diabetes-related chronic kidney disease: An analysis of the National Health and Nutrition Examination Survey, USA, 1999-2010Chia-Lin Lee(MD, PhD)^1, 2, 3,4^ ,Shang-Feng Tsai (MD, PhD) ^4, 5,6,^

Supplementary data

Table 1. Detail data regarding mortalities (per 1000 person-years) according to four levels of SUA.

| Uric acid (mg/dl) | All | 5-7 | 7-9 | 7-9 | >9 |
| --- | --- | --- | --- | --- | --- |
| N (%) | 1860 | 278 | 874 | 575 | 133 |
| mortality n(%)-weighted precentage |  |  |  |  |  |
| All-cause mortality | 649(29.68) | 91(25.44) | 290(28.1) | 212(32.6) | 56(39.42) |
| CVD mortality | 205(8.97) | 30(8.73) | 86(7.77) | 65(9.79) | 24(15.37) |
| Cancer mortality | 107(4.81) | 14(4.36) | 50(4.43) | 34(5.61) | 9(5.12) |
| CVD or cancer death | 312(13.78) | 44(13.09) | 136(12.2) | 99(15.4) | 33(20.5) |
| per 1000 person-years - weighted |  |  |  |  |  |
| All-cause mortality | 57 | 48 | 53 | 64 | 74 |
| CVD mortality | 17 | 16 | 15 | 19 | 29 |
| Cancer mortality | 9 | 8 | 8 | 11 | 10 |
| CVD or cancer death | 26 | 25 | 23 | 30 | 38 |

**Figure 1. Hazard ratio for all-cause, CVD related, cancer related, and CVD or cancer related mortality in different baseline level of SUA.**

**1A.Patients with non-DM CKD, with or without baseline CVD**


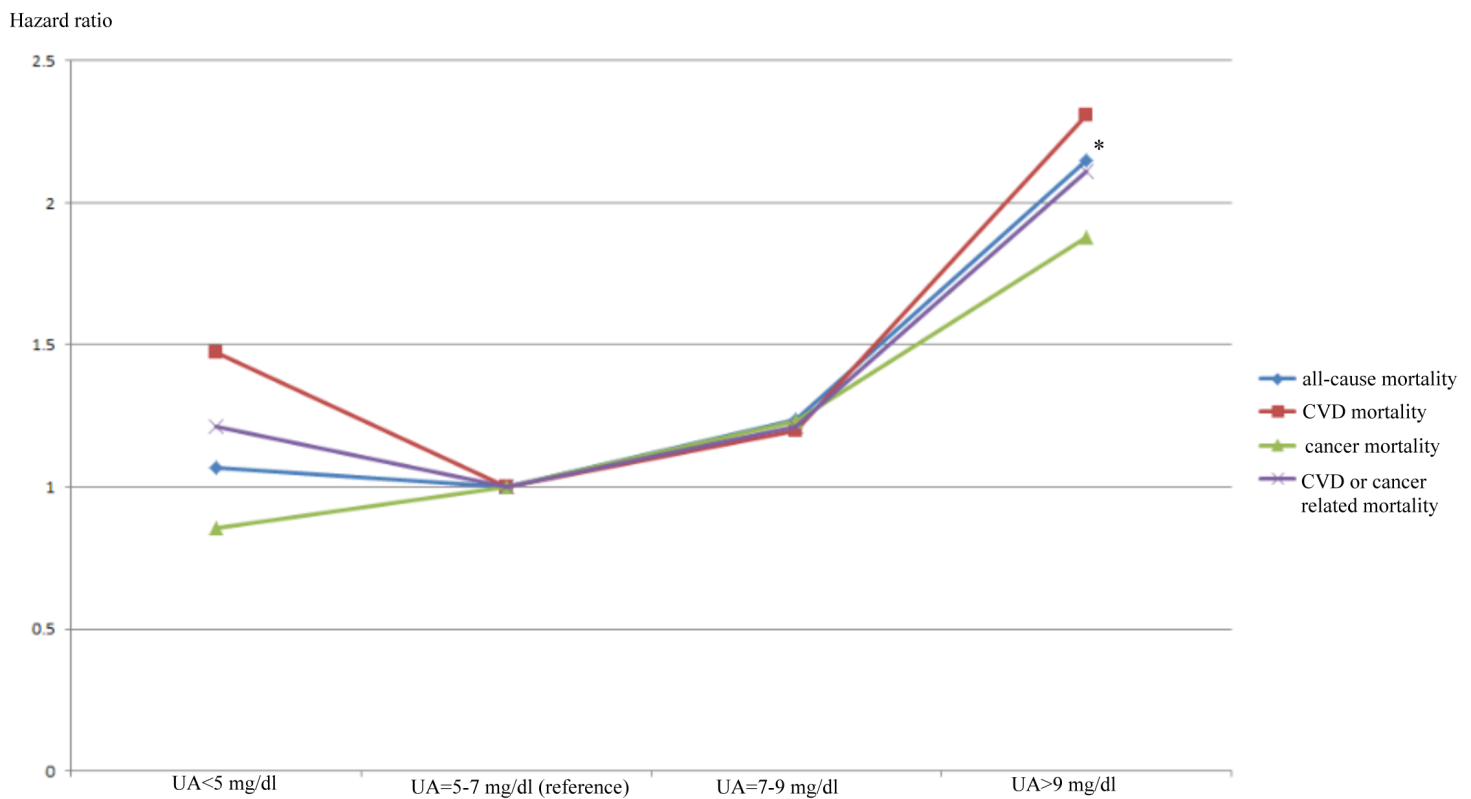


*: compared to SUA=5-7 mg/dl, p<0.05

**1B. Patients with non-DM CKD, without baseline CVD**


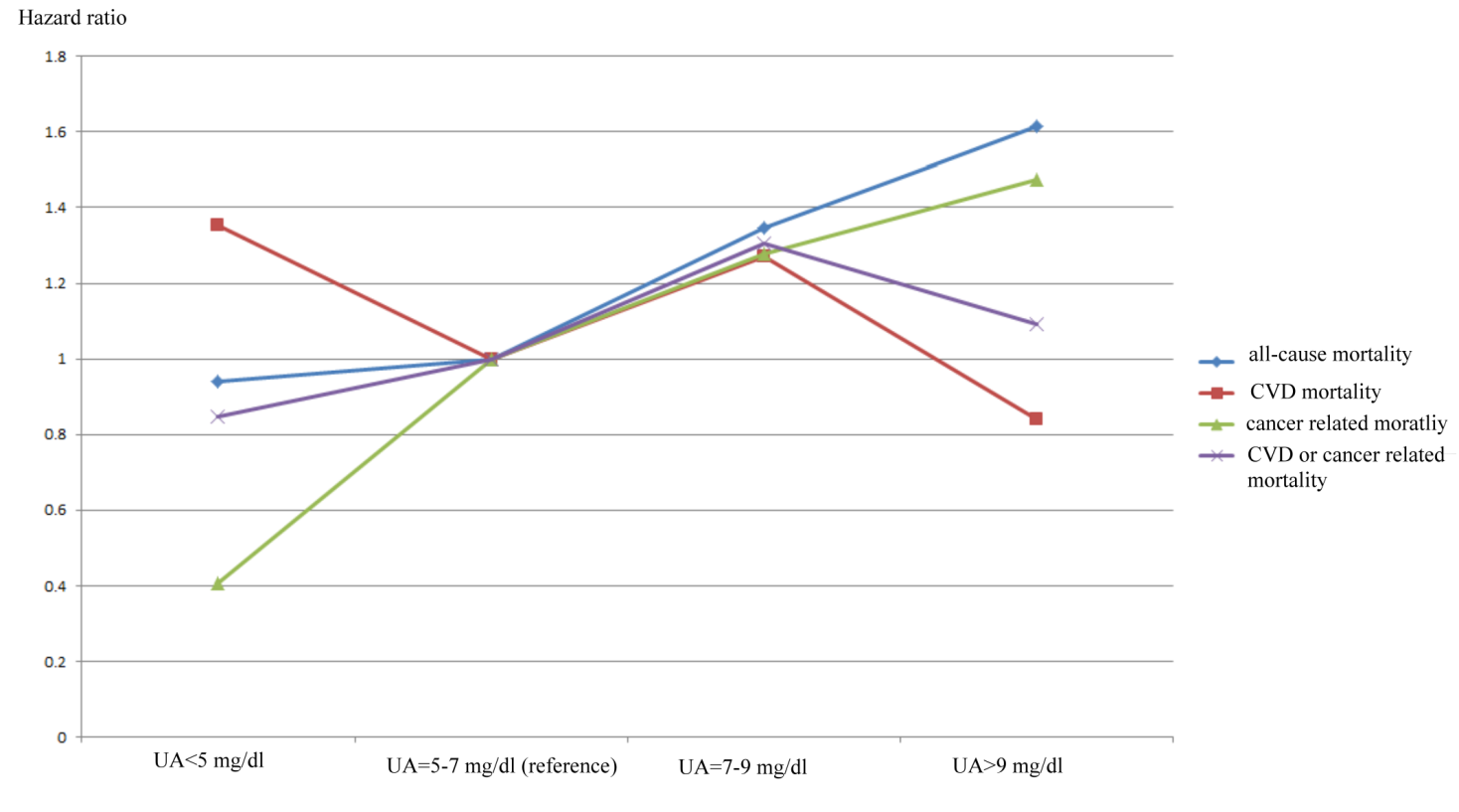


*: compared to SUA=5-7 mg/dl, p<0.05

**1C. Patients with non-DM CKD, with baseline CVD**


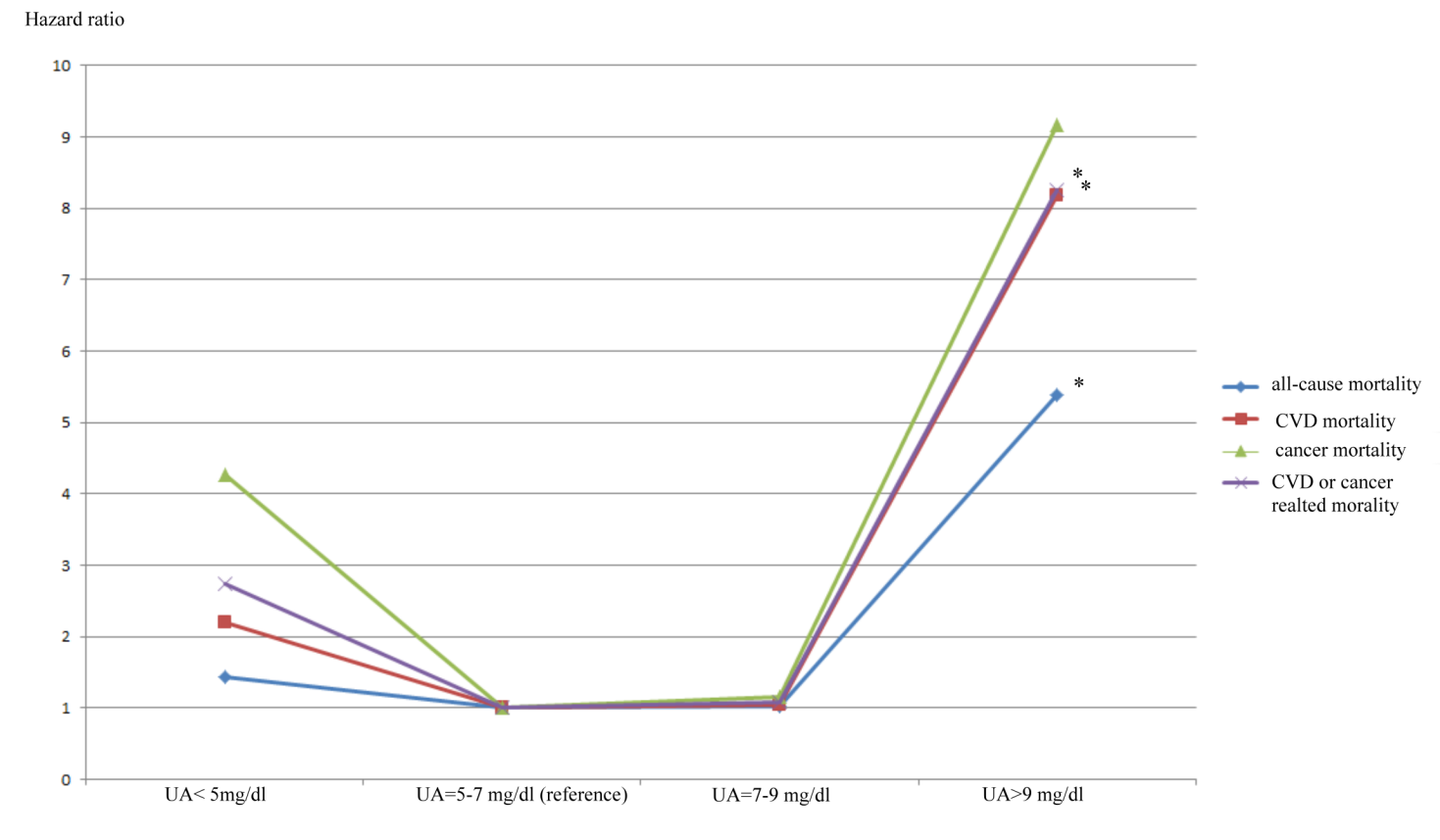


*: compared to SUA=5-7 mg/dl, p<0.05

**Figure 2. Hazard ratio for all-cause, CVD related, cancer related, and CVD or cancer related mortality in different baseline level of SUA based on CVD history or not.**

**(2A-2D: with or without CVD history; 2E-2H: without CVD history; 2I-2L: with CVD history)**

**2A. All-cause mortality in non-DM CKD patients, with our without CVD history**


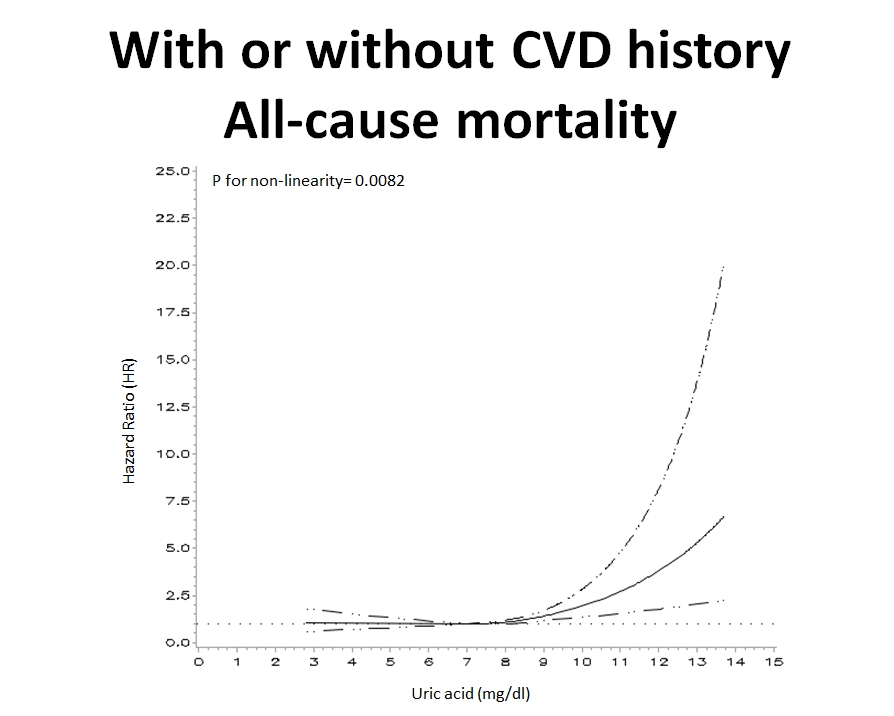


**2B. CVD mortality in non-DM CKD patients, with or without CVD history**


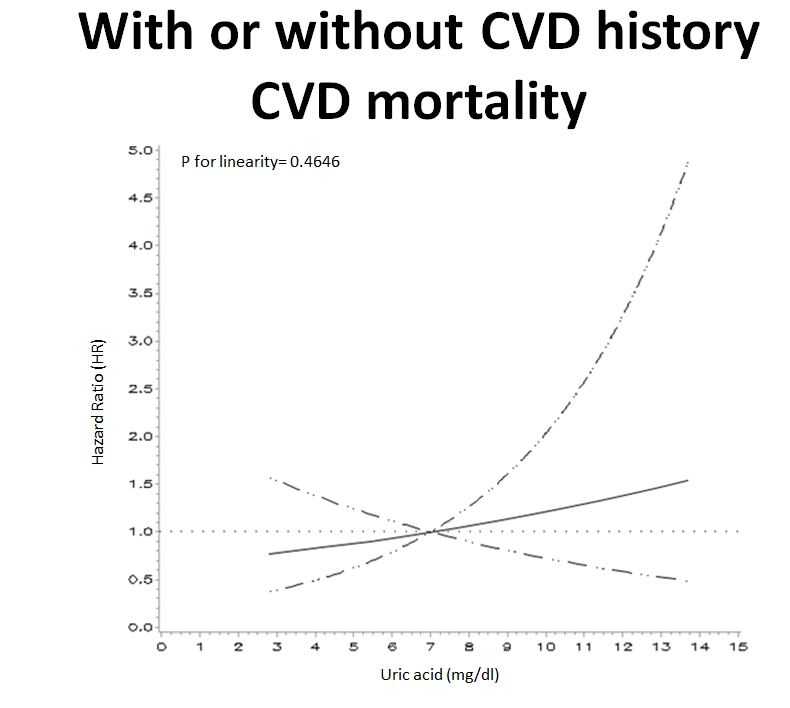


**2C. Cancer related mortality in non-DM CKD patients, with or without CVD history**


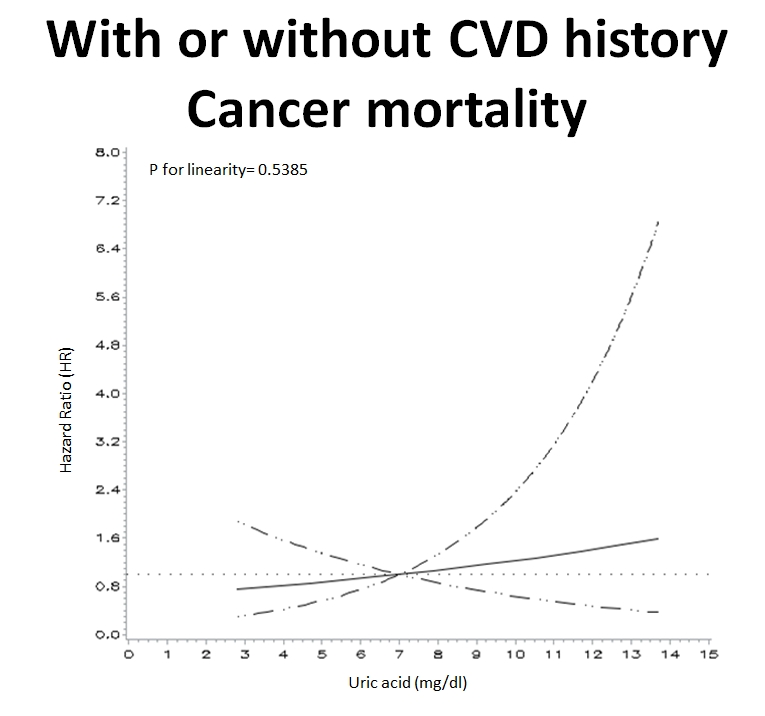


**2D. CVD or cancer related mortality in non-DM CKD patients, with or without CVD history**


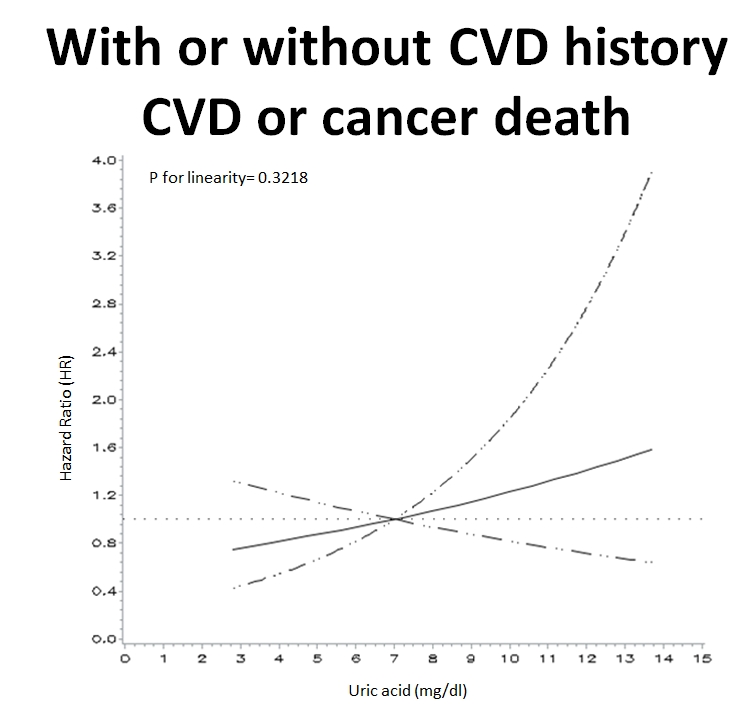


**2E. All-cause mortality in non-DM CKD patients, without CVD history**


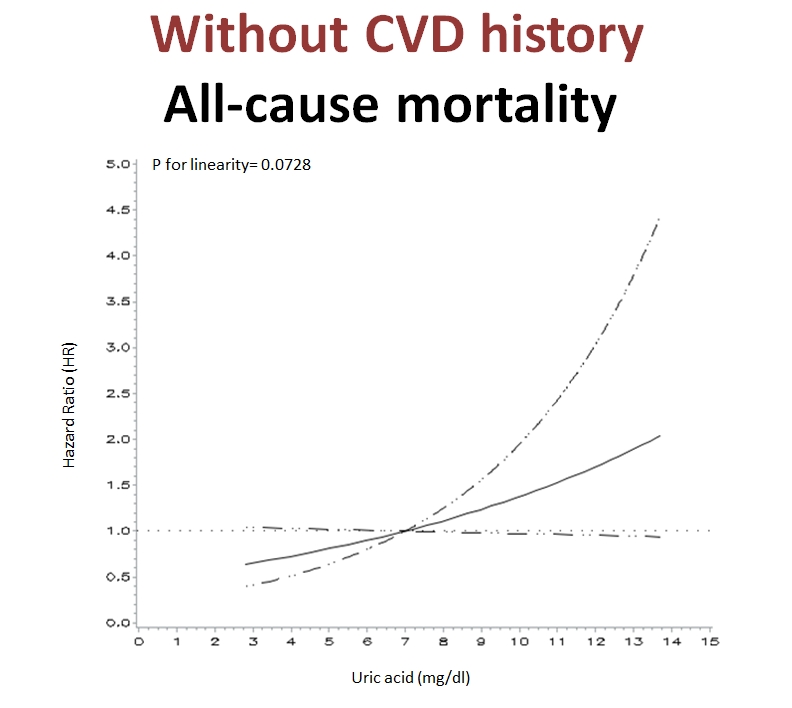


**2F. CVD mortality in non-DM CKD patients, without CVD history**


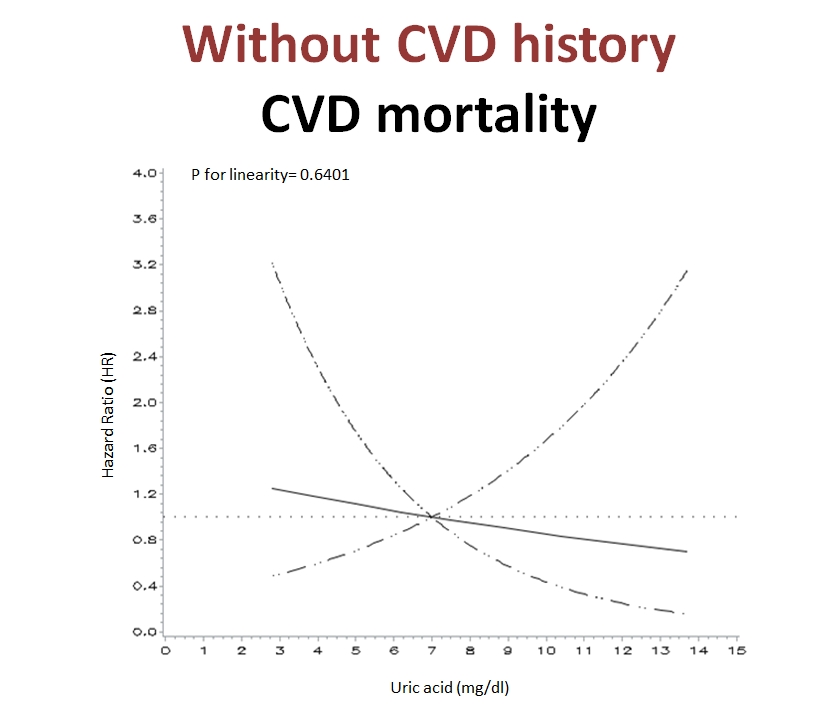


**2G. Cancer related mortality in non-DM CKD patients, without CVD history**


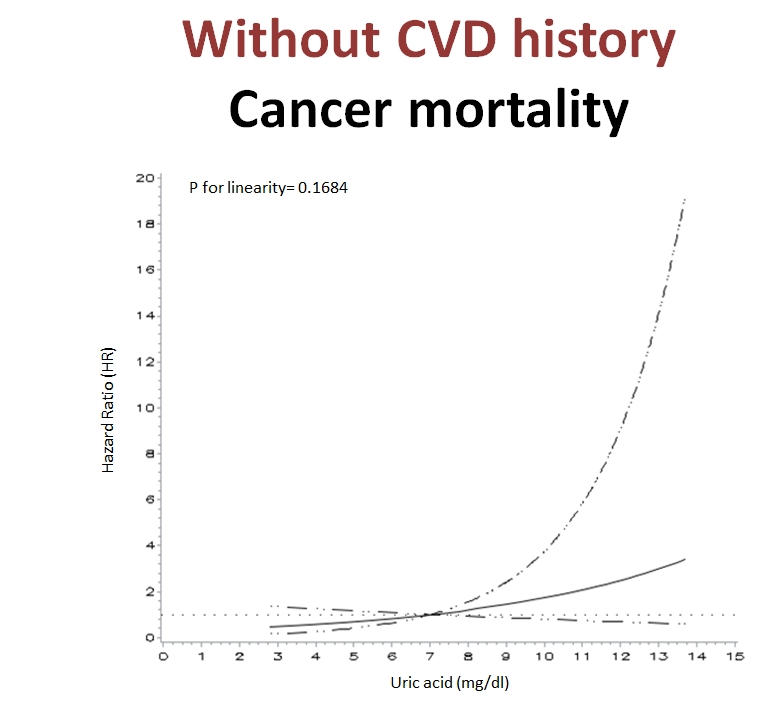


**2H. CVD or cancer related mortality in non-DM CKD patients, without CVD history**


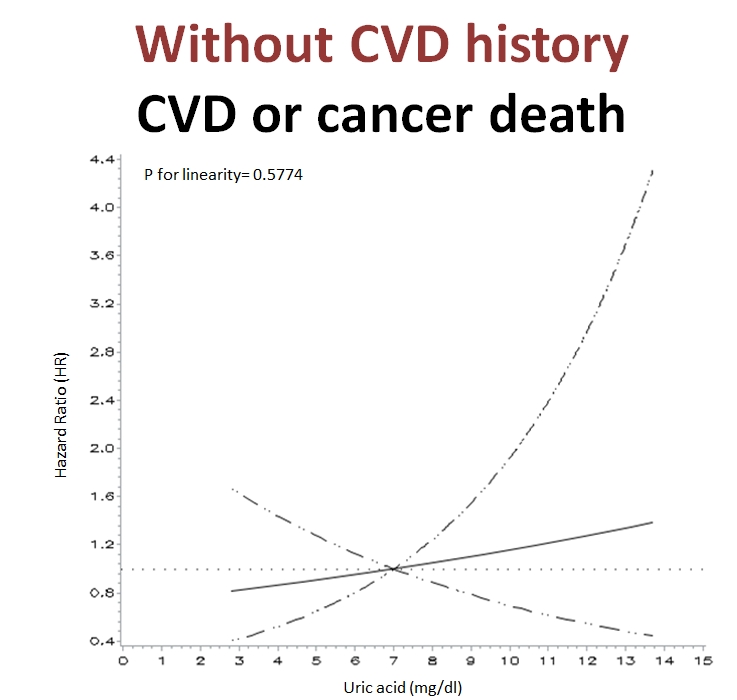


**2I. All-cause mortality in non-DM CKD patients, with CVD history**


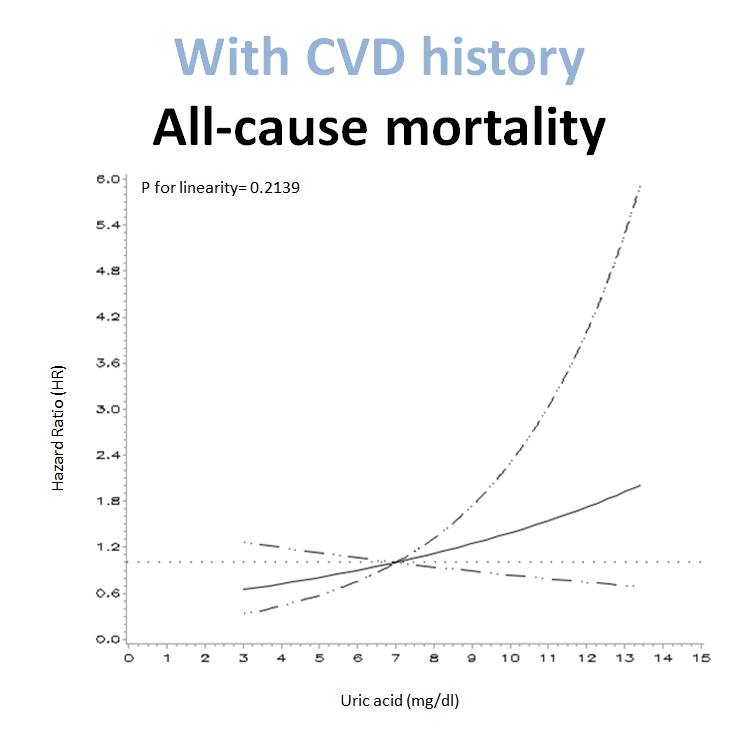


**2J. CVD mortality in non-DM CKD patients, with CVD history**


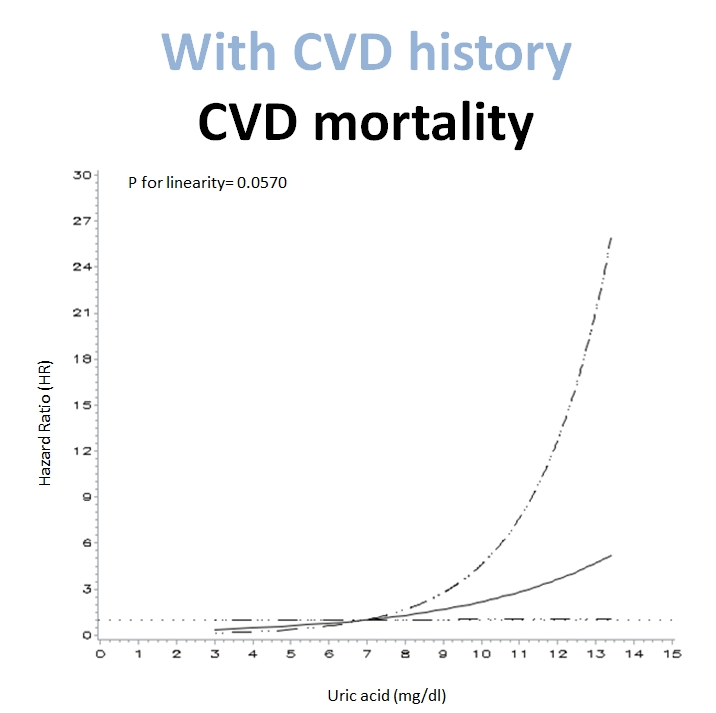


**2K. Cancer related mortality in non-DM CKD patients, with CVD history**


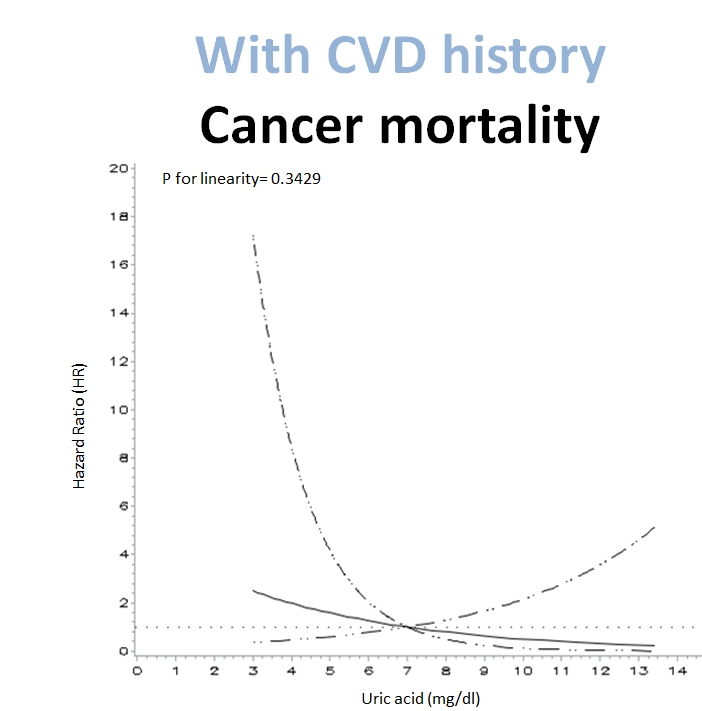


**2L. CVD or cancer related mortality in non-DM CKD patients, with CVD history**


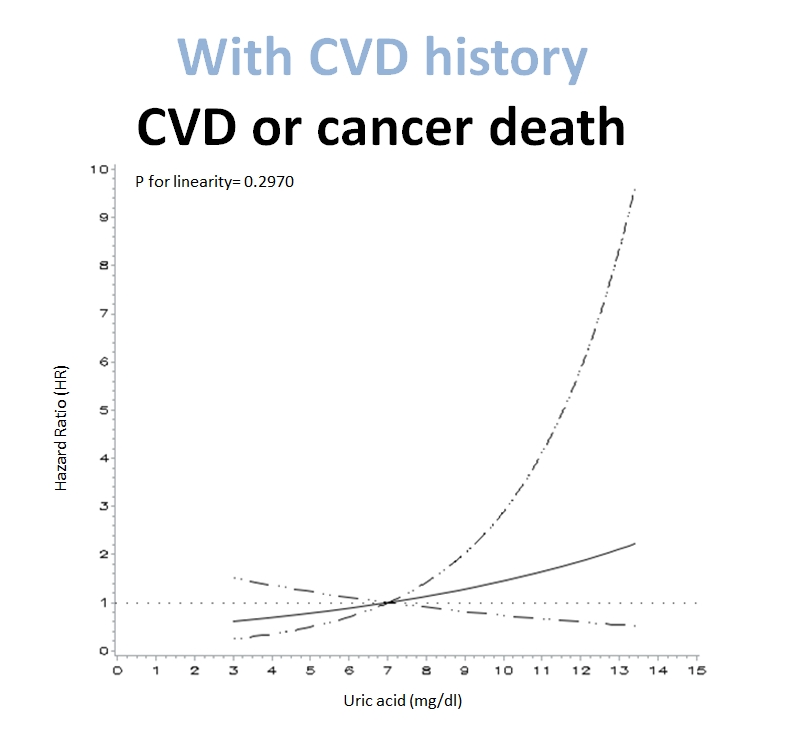

Supplement: Supplementary file 1 — Supplementary file1 [file 41598_2020_74747_MOESM1_ESM.docx]
